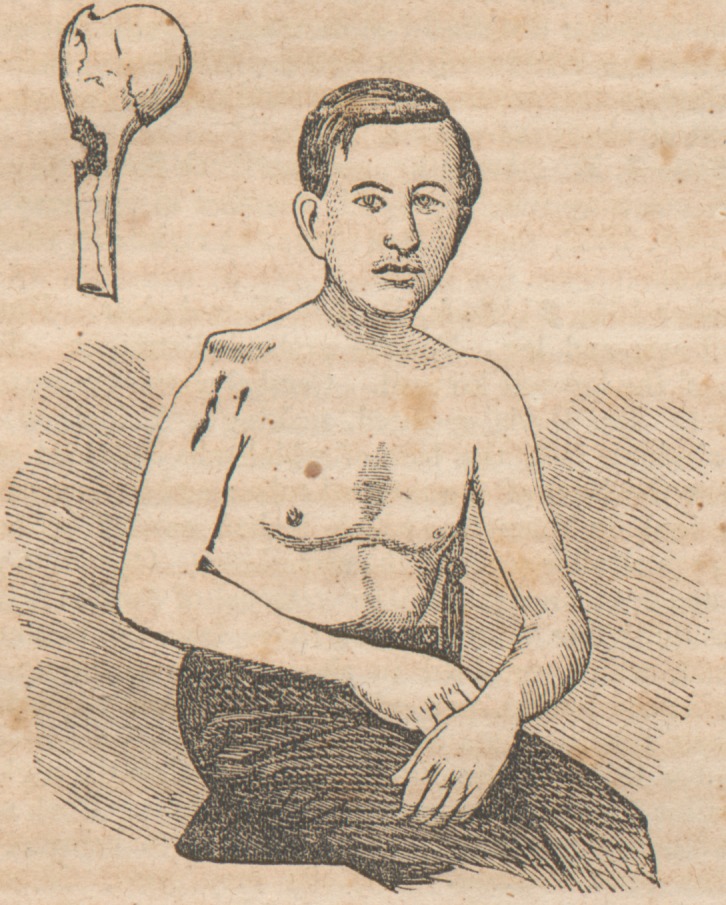# Excision of the Superior Third of Humerus

**Published:** 1864-03

**Authors:** Otis Frederic Manson

**Affiliations:** Surgeon P. A. C. S., in charge of General Hospital 24, Richmond, Va.


**Art. VIII.**—*Excision of the Superior Third of Humerus.* By Otis Frederic Manson, Surgeon P. A. C. S., in charge of General Hospital No. 24, Richmond, Va.

W. H. Ricketts, aged 22, private Co. "A," 13th Virginia regiment, was wounded in the battle of Gaines' Mill, on the 27th of June, 1862, by a Minie ball passing through the right arm near the shoulder joint, lacerating the capsule and splitting the humerus from its surgical neck, downwards, into many fragments to the extent of three inches.

He was admitted into the hospital, June 28th. Symptoms of great vital depression existing, operative procedure was postponed until the 6th of July.

*Operation.*—Complete anæsthesia was induced by the inhalation of chloroform.

The operation simply consisted in making a single perpendicular incision through the deltoid, from the acromion, downwards, to the extent of five inches. The bone was carefully exsected as far down as it was found to be injured, at which point a chain-saw was passed around and the shaft divided.

Using the longest of the fragmentary ends as a lever, the head of the bone was lifted and dissected from its socket.

Many spiculæ of bone were found imbedded in the surrounding muscles—these were carefully removed; the parts brought together by suture; the limb supported by pillows; and cold water dressing applied.

The cure of the case was greatly delayed by three separate and distinct attacks of erysipelas, occurring on the 13th and 26th of July, and the 22d of August, which were, however, promptly arrested, by the free use of sulphate of quinia and the tincture of the chloride of iron.

The patient has now very good use of his arm—the functions of the fore-arm and fingers being almost perfect. He writes a beautiful hand, and altogether, presents another of the many proofs of the value of this surgical expedient, in preference to that formerly practised in such cases—amputation at the shoulder joint

## Figures and Tables

**Figure f1:**